# Supplementary material for: Parallel wavelength-division-multiplexed signal transmission and dispersion compensation enabled by soliton microcombs and microrings
Source: Nat Commun. 2024 Apr 29;15:3645. doi: 10.1038/s41467-024-47904-2 (PMC11058204; doi:10.1038/s41467-024-47904-2)
Supplement: Supplementary file 1 — Supplementary Information [file 41467_2024_47904_MOESM1_ESM.pdf]

Supplementary Information for Parallel Wavelength-Division-Multiplexed Signal Transmission and Dispersion Compensation Enabled by Soliton Microcombs and Microrings

Supplementary Note 1: Program-controlled scheme for the single-soliton generation and stabilization

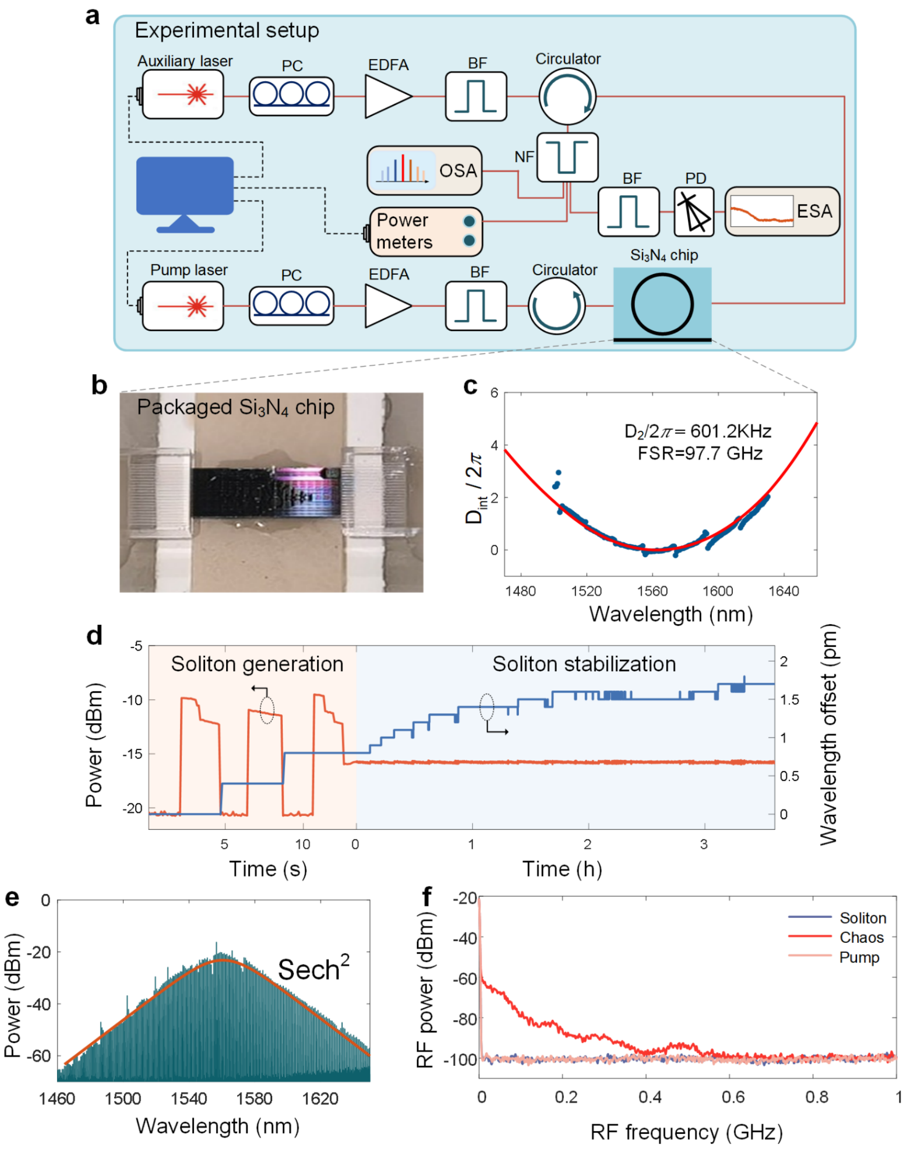

Supplementary Fig. 1. Program-controlled single-soliton microcomb generation and stabilization. a Experimental setup for the single-soliton generation and stabilization. PC: polarization controller; EDFA: erbium-doped fiber amplifier; BF: bandpass filter; NF: notch filter; OSA: optical spectrum analyzer; ESA: electric spectrum analyzer; PD: photodetector. **b** Photograph of the packaged Si_3N_4 chip. **c** Integrated dispersion of the adopted TE_0 mode. The $D_2/2\pi$ is 601.2 kHz and the FSR is 97.7 GHz. **d** Comb power and auxiliary laser wavelength variation traces during the single-soliton generation and stabilization process. We monitor a small portion of the comb power through the 1:9 splitter. **e** Spectrum of the single soliton with a smooth sech^2 envelope. **f** RF spectrum at low RF frequency when we filter out the single line to the PD of the single soliton, chaos combs, and pump laser.

We implemented a program-controlled scheme based on the red-detuned pump entrance forward-tuning method with the assistance of an auxiliary laser to generate the long-term single-soliton microcomb[1]. The thermal lock effect of the auxiliary laser on the blue-detuned side can ensure the pump laser to approach the soliton state. The schematic experimental setup is shown in Supplementary Fig. 1a. Two counter-propagating optical waves from the pump and from the auxiliary lasers were coupled into the silicon nitride (Si_3N_4) microresonator. We utilized an optical circulator to separate the pump light and the auxiliary light. Meanwhile, we utilized a notch filter to filter out the pump light and the reflective auxiliary light. Then, we recorded the optical spectra during the comb evolution with an optical spectrum analyzer (OSA) and characterized the low-frequency noise property by using an electrical spectrum analyzer (ESA). We measured the comb power during the tuning process to determine the generation of the single soliton. The output powers of the pump and of the auxiliary laser were set to 31 dBm and 32 dBm and the coupling loss is ~3 dB/facet after the packaging, as shown in Supplementary Fig. 1b. Supplementary Fig. 1c depicts the integrated dispersion curve of the adopted TE_0 mode. The TE_0 mode has an anomalous dispersion[2] with the $D_2/2\pi$ of 601.2 kHz and a free spectral range (FSR) of 97.7 GHz. The single soliton can be generated with a high probability only when the auxiliary laser supports an efficient thermal compensation[1].

We adopted the iteration method to generate the single soliton and the tuning process is shown in Supplementary Fig. 1d. These two traces represent the variations of the comb power and of the wavelength offset of the auxiliary laser during the generation and the stabilization process. During the soliton generation, we performed the forward scanning with the red-detuned entrance for the pump laser, and the auxiliary laser wavelength was fixed during the pump tuning process. If a single soliton is not generated, the auxiliary laser wavelength is increased by 0.4 pm, and the pump scanning process restarts. After several iterations, we can deterministically generate the single soliton, and we begin the stabilization procedure. The pump laser wavelength was fixed during the soliton stabilization process. We tuned the auxiliary laser wavelength to vary the intracavity power of the auxiliary laser, and the position of the pump resonance changed accordingly due to the thermal effect and to the cross-phase modulation (XPM) of the auxiliary laser under a high power. Therefore, we precisely tuned the auxiliary laser wavelength to lock the comb power to the value when the single soliton was initially generated. In the experiment, the single soliton was stabilized for more than 3 hours. Supplementary Fig. 1e shows the recorded spectrum of the single soliton with a smooth $sech^2$ envelope. Compared to the chaotic comb, the single soliton has a lower noise which is similar to that of the pump laser, as shown in Supplementary Fig. 1f.

Supplementary Note 2: Simulations of the reconfigurable MRRs-based chromatic dispersion compensator (CDC)

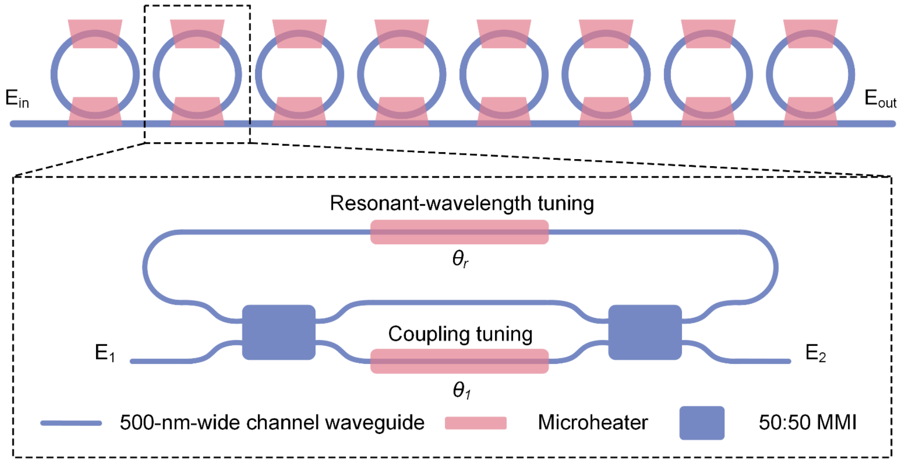

Supplementary Fig. 2. Schematic structure of the CDC. Inset: Enlarged schematic structure of the MRR. MMI: multimode interferometer.

We designed the CDC using the transfer matrix method. Supplementary Fig. 2 shows the schematic structure of the CDC. We express the complex amplitude transmission of the ith coupling-tunable microring resonator (MRR) $T_{single}(i)$ as:

$$T_{single}(i) = \frac{E_2}{E_1} = je^{-j(\theta_{1_i}+\theta_{r_i})} \cdot \frac{e^{j(\frac{\theta_{1_i}}{2}+\theta_{r_i})}\sin\left(-\frac{\theta_{1_i}}{2}\right)+j\alpha_i e^{-j\phi_i}}{1+j\alpha_i e^{-j(\frac{\theta_{1_i}}{2}+\theta_{r_i})}\sin\left(-\frac{\theta_{1_i}}{2}\right)e^{-j\phi_i}} \quad (1)$$

where E_2 and E_1 are the complex amplitudes of the electric fields of the output and the input of the MRR, θ_{1_i}, θ_{r_i}, α_i, and ϕ_i represent the phase shift of the coupler, the phase shift of the ith MRR, the amplitude loss factor of the ith MRR, and the round-trip phase of the ith MRR. Here $\phi_i = 2\pi n_{eff} L_i/\lambda$, where n_{eff} is the effective refractive index of the waveguide in the MRR, L_i is the round-trip length of the MRR, and λ is the optical wavelength in a vacuum. All the 8 MRRs share the same design. As we utilize the Mach-Zehnder interferometer (MZI) as the tunable coupler, the equivalent power coupling coefficient of the ith MRR is $K_{eqv_i} = cos^2(\theta_{1_i}/2 + \Delta\phi_{ini_i})$, where $\Delta\phi_{ini_i} = \phi_{up_i} - \phi_{down_i}$ is the initial phase difference (when $\theta_{1_i} = 0$) between the two arms of the MZI, and ϕ_{up_i} and ϕ_{down_i} are the phase changes induced by the upper and the lower arms. We assume $\Delta\phi_{ini_i} = 0\ (i = 1, 2, \ldots, 8)$ in our modelling. We then model each MRR as a standard laterally coupled MRR with a real waveguide-MRR power coupling coefficient K_{eqv} (assuming no coupling loss) and with a resonator phase shift θ_r. The complex amplitude transmission of the ith MRR is:

$$T_{single}(i) = \frac{\sqrt{1-K_{eqv_i}} - \alpha_i e^{-j(\phi_i+\theta_{r_i})}}{1-\alpha_i\sqrt{1-K_{eqv_i}}\, e^{-j(\phi_i+\theta_{r_i})}} \quad (2)$$

We calculate the amplitude transmission of the eight cascaded MRRs by multiplying the amplitude transmission of each MRR as follows:

$$T_{cascaded} = \prod_{i=1}^{8} T_{single}(i) \quad (3)$$

Then, we obtain the phase and group delay responses of the cascaded MRRs as:

$$\emptyset = arctan\left(\frac{Im(T_{cascaded})}{Re(T_{cascaded})}\right) \quad (4)$$

$$\tau = -\frac{d\emptyset}{d\omega} \quad (5)$$

where $\emptyset$ is the phase response, $Im(\cdot)$ and $Re(\cdot)$ represent the imaginary and the real parts, respectively, τ is the group delay response, and ω is the optical angular frequency. We obtain the chromatic dispersion (CD) parameter D as follows:

$$D = \frac{d\tau}{d\lambda} \quad (6)$$

With the theoretical model (Eqs. (1) – (6)), we utilized a nonlinear optimization method based on the sequential quadratic programming (SQP) algorithm to optimize the real power coupling coefficient K_{eqv} and the phase shift θ_r of each MRR (K_{eqv} and θ_r are assumed independent parameters) to attain the desired CD compensation for different lengths of single-mode fibers (SMFs) with an operation bandwidth of 32 GHz and with a CD ripple of less than ±10 ps/nm. We assumed a waveguide transmission loss of 4 dB/cm in the simulations. In the simulation for 8 MRRs, we have 16 independent parameters (power coupling coefficient K_{eqv} and phase shift θ_r for each ring) to adjust to realize the target. We monitored the ripples of the calculated CD values within the desired bandwidth in each iteration and adjusted all the 16 independent parameters of 8 MRRs to minimize the ripples. The power coupling coefficients are constrained in the range of 0 to 1 while we impose no constraints for the phase shifts.

We set the target of the optimization to be a constant compensated CD value D_{tar} of the corresponding lengths of SMFs within the 32-GHz bandwidth. We define a figure-of-merit (FOM) as $FOM = 1/n \cdot \sum_{i=1}^{n}(D_i - D_{tar})^2$, where n is the number of CD values sampled within the bandwidth and D_i is the simulated CD value at a certain wavelength. For example, if we consider the bandwidth to be 0.2 nm, and the wavelength increment is 1 pm, then n is 201, D_i is the ith sampled point of the simulated CD. The FOM reflects the variance of the CD within the bandwidth with respect to the target CD parameter. The optimization is reached when the FOM is minimized. As different numbers of MRRs are required for the CD compensation of various lengths of SMFs, we started from one single MRR and more MRRs were introduced one at a time. If the FOM is minimized, the number of MRRs is confirmed. If not, we add one more MRR into the optimization and implement the optimization again until we satisfied the FOM of the optimization. The optimization of the CD compensation for a 20-km-long SMF within a 50-GHz bandwidth is the same except for a larger preset bandwidth.

A total of 4, 5, 7, and 8 MRRs are required for CD compensation of the 4 lengths of SMFs in a 32-GHz bandwidth while 8 MRRs are needed for a 20-km-long SMF within a 50-GHz bandwidth. We simulate the group delay responses for CD compensation of 40 km SMF across a large wavelength span ranging from 1540 nm to 1580 nm, using a linear changed coupling coefficient upon different wavelength channels. In the simulation, we also consider the material and structure dispersion (i.e., the effective refractive index of the mode varies with wavelength). Supplementary Figs. 3a to 3c show the simulated group delay responses with the power coupling coefficient variation slope of 0.0005/nm, 0.002/nm, and 0.004/nm, respectively. The power coupling coefficient is larger at longer wavelengths. The use of MZI tunable coupler instead of a directional coupler in the MRR has less coupling coefficient variation over the operation bandwidth. However, the insertion loss and phase response of the multimode interferometers (MMIs), the propagation loss of the waveguides, and the thermo-optic phase shifts are all wavelength-dependent, which results in wavelength-dependent coupling coefficients, thus affects the envelope of the group delay response. Supplementary Fig. 3d

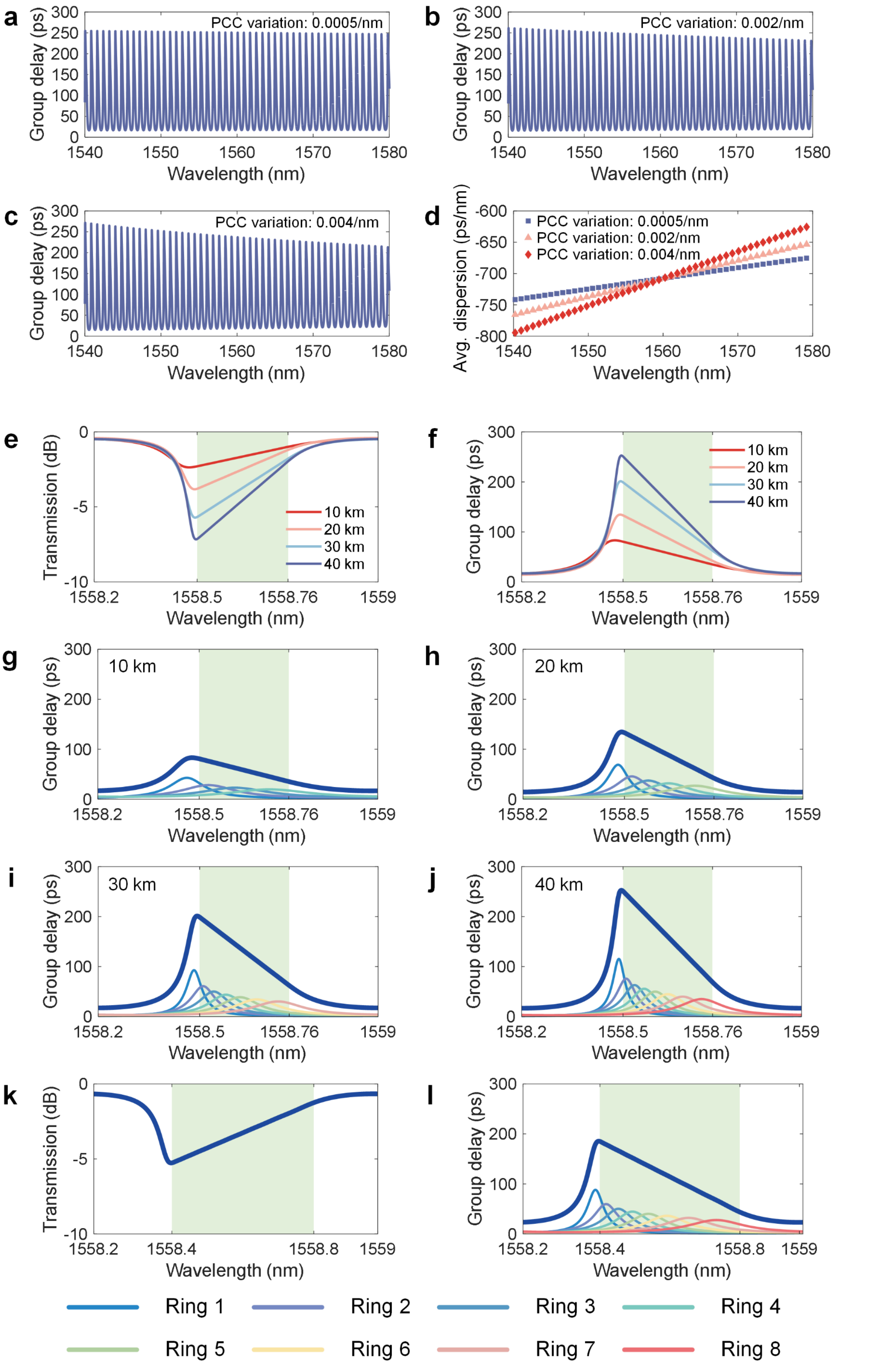

Supplementary Fig. 3. Simulations of the MRRs-based CDC. a, b, c Simulated group delay responses of the CD compensation of 40 km SMF within a bandwidth of 32 GHz with a power coupling coefficient slope of **(a)** 0.0005/nm, **(b)** 0.002/nm, and **(c)** 0.004/nm. PCC: power coupling coefficient. **d** Extracted average CD of each

channel under the three PCC variation slopes. **e, f** Simulated **(e)** transmission spectra and **(f)** group delay responses at the wavelength around 1558.7 nm for the CD compensation of 10 km, 20 km, 30 km, and 40 km SMFs. **g-j** Simulated group delay responses of each MRR and their summation of CD compensation for **(g)** 10 km, **(h)** 20 km, **(i)** 30 km, and **(j)** 40 km SMF transmission. **k, l** Simulated **(k)** transmission and **(l)** group delay responses of CD compensation for 20-km-long SMF within a 50-GHz bandwidth.

shows the extracted average CD of each channel under the three power coupling coefficient variation slopes. The average CD is calculated as $D_{avg} = (\tau_1 - \tau_2)/(\lambda_1 - \lambda_2)$, where λ_1 and λ_2 are the shorter and longer wavelength ends across the operation bandwidth of each channel, τ_1 and τ_2 are the corresponding group delays, respectively. We can see that the average CD vary linearly with the wavelength. The variation of the envelope of the group delay response is larger with a larger power coupling coefficient variation, which also leads to a larger CD variation across the wavelength span. Under the power coupling coefficient variation slope of 0.004/nm, the average CD ranges from approximately -798 ps/nm to -622 ps/nm within the 40-nm wavelength span.

Supplementary Table 1. Simulated resonant wavelengths and group delays of the MRRs

Length of SMF (km)	Bandwidth (GHz)	MRR No. 1	2	3	4	5	6	7	8
10	32	0	0.059	0.131	0.225	/	/	/	/
		45.96	30.37	24.64	20.96				
20	32	0	0.037	0.082	0.137	0.209	/	/	/
		75.28	50.09	40.97	34.85	29.43			
30	32	0	0.024	0.052	0.085	0.123	0.169	0.227	/
		103.55	68.43	56.70	49.65	44.01	38.82	33.66	
40	32	0	0.018	0.039	0.064	0.092	0.124	0.163	0.213
		143.87	96.61	81.52	72.47	65.31	58.67	51.85	44.22
20	50	0	0.029	0.062	0.100	0.143	0.193	0.252	0.328
		97.94	66.76	56.47	50.26	45.31	40.71	35.99	30.72

Note: The numerical data in the upper row in each grid represent the relative resonant wavelengths of each MRR with a unit of nm while those in the lower row stand for the group delay at the resonant wavelength of each MRR with a unit of ps.

The simulated transmission and group delay responses over one FSR at the wavelength around 1558.7 nm for CD compensation within the 32-GHz bandwidth are shown in Supplementary Figs. 3e and 3f. The group delay varies inversely with the wavelength in the operation bandwidth while the CD compensation of a longer SMF exhibits a larger absolute slope. The group delay responses of each MRR and their summation for different lengths of SMFs are illustrated in Supplementary Figs. 3g to 3j. The required maximum group delays are all positive, and they are smaller for the MRRs resonant at the longer wavelength side, indicating that all the MRRs are working in the over-coupling regime and the power coupling coefficients are higher for the MRRs resonant at the longer wavelength side. Supplementary Figs. 3k and 3l show the transmission and group delay responses of CD compensation for the 20-km-long SMF within a 50-GHz bandwidth. The simulated resonant wavelengths and group delay responses of the MRRs for CD compensation of the 10-, 20-, 30-, and 40-km-long SMFs in the 32-GHz bandwidth and of the 20-km-long SMF in the 50-GHz bandwidth are listed in Supplementary Table 1. The numerical data in the upper and lower rows of each grid

stand for the resonance wavelength and the group delay of the corresponding MRR, respectively. The resonant wavelengths are relative to that of the first MRR. The numerical data shown in Supplementary Table 1 demonstrate that when the MRRs are tuned to the desired wavelengths with the corresponding desired group delay, we obtain a flat in-band CD response.

Supplementary Note 3: Calibration of the CDC

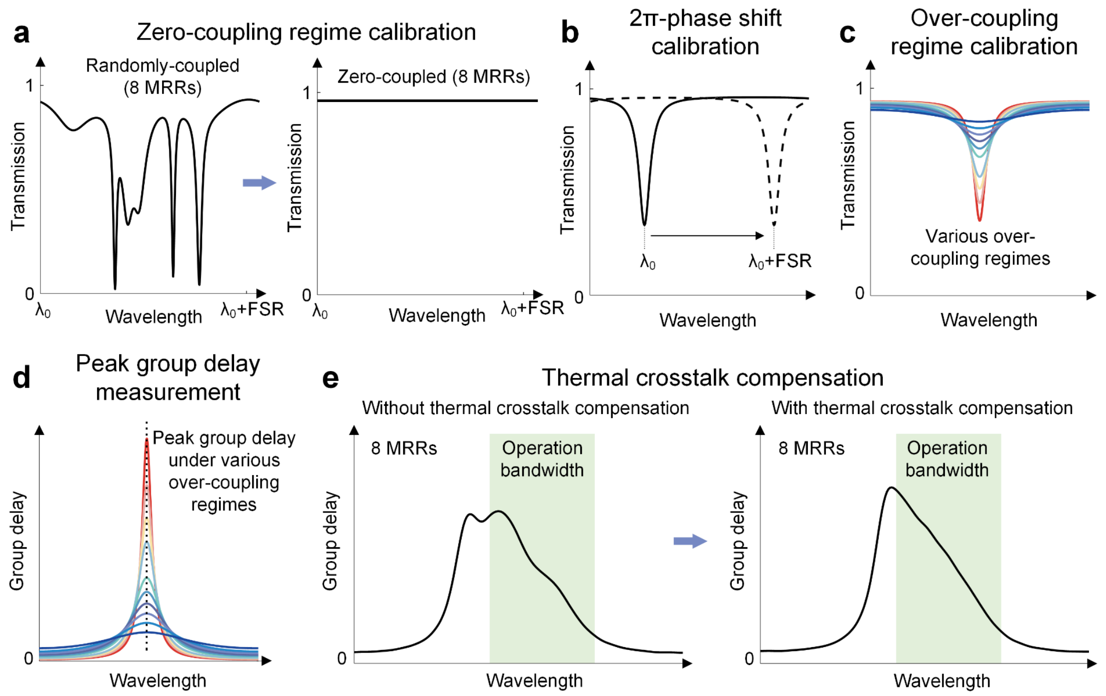

Supplementary Fig. 4. Schematics of the line shapes in various calibration steps. a Left: Schematics of the transmission under randomly coupled conditions for 8 MRRs. Right: Schematics of the transmission after zero-coupling regime calibration. **b** Schematics of the transmission of 2π-phase shift calibration. **c** Schematics of the transmission of over-coupling regime calibration. **d** Schematics of the group delay of the peak group delay measurement under various over-coupling regimes. **e** Left: Schematics of the group delay of 8 MRRs without thermal crosstalk compensation. Right: Schematics of the group delay of 8 MRRs with thermal crosstalk compensation.

Calibration of MRRs is essential in obtaining the best-performance CDC as all the MRRs are in coupling regimes and resonant wavelengths that generally deviate from their designed characteristics when no control voltages or algorithms are applied. As shown in Supplementary Fig. 4, we divide the calibration protocol into 5 steps: **(1)** the zero-coupling regime calibration of 8 MRRs (Supplementary Fig. 4a), **(2)** the 2π-phase shift calibration of the resonant wavelength of each MRR (Supplementary Fig. 4b), **(3)** the over-coupling regime voltage range calibration of each MRR (Supplementary Fig. 4c), **(4)** the group delay measurement under various over-coupling regimes of each MRR (Supplementary Fig. 4d), and **(5)** the thermal crosstalk compensation (TCC) of 8 MRRs (Supplementary Fig. 4e). We first implemented the zero-coupling calibration because in the following calibrations, we tuned the MRRs one by one, and we must tune all the other MRRs to the zero-coupling regime except for the one under tuning. Then, we calibrated the voltage for a 2π-phase shift of the resonant wavelength of each MRR to allow us to find the applied voltage needed for the resonant wavelength-tuning phase shifter of each MRR to tune the resonant wavelength. Next, as we tune each MRR according to the data shown in Supplementary Table 1, we need to obtain the group delay of each MRR under various over-coupling regimes. Finally, as we observed a severe thermal crosstalk-induced distortion of the measured responses after the (1) – (4) calibration steps, we further implemented the TCC to eliminate the influence of the thermal crosstalk. The detailed calibrations of each step are discussed in the following sections.

Firstly, each MRR was tuned to the zero-coupling regime by minimizing the resonant notches in

the transmission spectrum to obtain the required voltage for the zero-coupling operation. Although the calibration objective is rather simple and clear, the transmission spectra of the zero- and unit-coupling regimes are almost identical except for the slightly different insertion losses, which makes it hard to distinguish between these two states by only referring to one flat transmission spectrum. As the resonant peaks are generally narrower and sharper in the under-coupling regime which is close to zero-coupling while they are wider and smoother in the over-coupling regime, we swept the applied voltage V_{i-cpl} $(1 \leq i \leq 8)$ on the phase shifter of the MRR coupler (the coupling-tuning phase shifter, as shown in Supplementary Fig. 2) one by one from 0 to 6 V with a step of 0.1 V, and measured the corresponding transmission spectra to identify the zero-coupling regime from the spectral lineshape. Supplementary Figs. 5a and 5b show the normalized transmission spectra before and after the zero-coupling regime calibration. We obtained an insertion loss of ~6.2 dB, corresponding to an average insertion loss of 0.75 dB for each MRR. The first row of Supplementary Table 2 lists the required voltages for the zero-coupling regime of each MRR.

Supplementary Table 2. Configuration parameters of the MRRs

	MRR No.							
	**1**	**2**	**3**	**4**	**5**	**6**	**7**	**8**
**Zero-coupling voltage (V)**	5.150	6.225	6.050	6.050	6.050	5.725	6.150	6.050
**2π-phase shift voltage (V)**	5.575	5.750	5.800	5.750	5.750	5.600	5.875	5.775
**Over-coupling voltage range (V)**	3.4~4.4	2.9~4	2.7~4.1	2.9~4.2	2.9~4.2	2.5~4	2.9~4	2.9~4.3
**FSR (nm)**	0.807	0.807	0.806	0.807	0.807	0.807	0.806	0.806

Next, we calibrated the 2π-phase shift voltages of each MRR by increasing the voltage V_{res} on the phase shifter in the MRR (the resonant wavelength-tuning phase shifter, as shown in Supplementary Fig. 2) until the resonance shifts by one FSR. The other MRRs were tuned to the zero-coupling regime. The second row of Supplementary Table 2 shows the measured 2π-phase shift voltages for the 8 MRRs, which are in the range of 5.575 V to 5.875 V, corresponding to a power consumption varying from 17.27 mW to 19.18 mW.

After that, we swept the voltages on the coupling-tuning phase shifters to obtain the voltage ranges for the over-coupling regime. We identified the over-coupling regime by referring to the transmission spectrum of the critical-coupling regime, which exhibits a maximum extinction ratio (ER) among all the operational conditions. The third row of Supplementary Table 2 illustrates the voltage ranges of the over-coupling regime of each MRR. The FSR of each MRR is also shown in Supplementary Table 2. During all the calibration steps, a thermo-electric cooler (TEC) was placed under the CDC chip to control the on-chip temperature, which was set to 20℃.

Next, we measured the group delay responses under the over-coupling regime to obtain the relationship between the peak group delay and the applied coupling voltages of all the MRRs. We tuned the MRR under test to realize various coupling coefficients in the over-coupling regime while measuring the corresponding group delay responses. During this procedure, we tuned the MRR one by one and the other MRRs were tuned to the zero-coupling regime. Supplementary Figs. 5c and 5d show the measured transmission and group delay responses of the first MRR under various

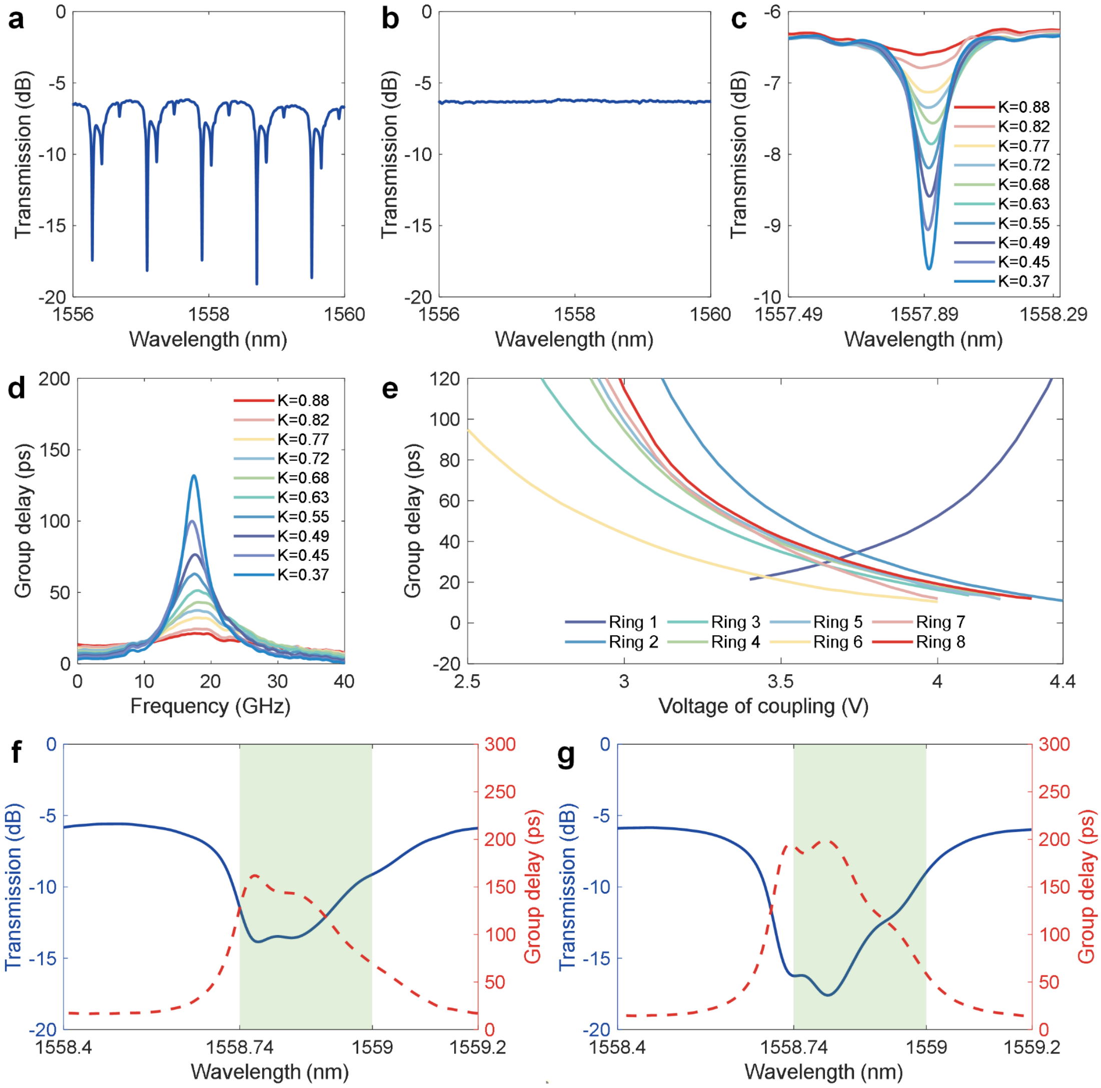

Supplementary Fig. 5. Experimental calibrations of the CDC. a, b Measured transmission spectrum **(a)** before and **(b)** after the zero-coupling regime calibration. **c, d** Measured **(c)** transmission spectra and **(d)** group delay responses of the first MRR with various power coupling coefficients under over-coupling regimes. **e** Measured maximum group delays with respect to the applied coupling voltages of 8 MRRs. **f, g** Measured transmission and group delay responses of CD compensation for **(f)** 30 km and **(g)** 40 km SMFs transmission without the TCC.

power coupling coefficients in the over-coupling regime. In this test, we fixed the resonant wavelengths of all the MRRs to the same wavelength by adjusting the resonant wavelength-tuning phase shifters upon varying the coupling coefficient. The extracted relationship between the peak group delay and the applied coupling voltage of each MRR is shown in Supplementary Fig. 5e. We attribute the different trend of the first MRR to the fabrication error-caused reverse coupling change of the MZI coupler. For the first MRR, K_{eqv_1} first increases with the increased θ_{1_1} (when the coupler voltage is under 3.4 V), and then decreases with the increased θ_{1_1} (when the coupler voltage is in the range of 3.4 V to 4.4 V), indicating that the initial phase difference of the MZI coupler is $\Delta\phi_{ini_1} < 0$. For the other MRRs, $\Delta\phi_{ini} > 0\ (i = 2, 3, \ldots, 8)$. The fabrication error causes the opposite sign of the initial phase difference of the first MRR.

Following the calibration results, we can tune all the MRRs to achieve the CD compensation

states for various lengths of SMFs. We first extracted the peak group delay and the corresponding resonant wavelengths of each MRR from the simulation, as shown in Supplementary Table 1. Then, we acquired the required voltages for the coupling tuning from the measured relationship between the peak group delay and the applied voltages on the couplers, as shown in Supplementary Fig. 5e, and then we applied the voltages to the MRRs under test. Next, we adjusted the resonant wavelength from the initial wavelength to the desired one. We measured the transmission spectrum in the range of 1556 nm to 1560 nm to extract the resonant peak wavelength λ_{1_i}, and then we calculated an applied voltage on the resonant wavelength-tuning phase shifter by:

$$V_{i-res} = \sqrt{\frac{\lambda_{0_i} - \lambda_{1_i}}{FSR_i} \cdot V_{i_{2\pi}}^2} \quad (7)$$

where λ_{0_i}, FSR_i, and $V_{i_{2\pi}}$ denote the desired resonant wavelength in free space, the free spectral range, and the 2π-phase-shift voltage for the resonant wavelength tuning of the ith MRR. We implemented several iterations (no more than 3) in this wavelength-tuning process. We note that all the eight MRRs were tuned one by one. While one MRR was tuned, the other MRRs were adjusted to the zero-coupling state with no applied voltages to the resonant wavelength-tuning phase shifters. After completing the calibration of each MRR, we applied the acquired voltages to all the MRRs together to achieve the desired CD compensation. The measured transmission and group delay responses for CD compensation of 30- and 40-km-long SMFs are shown in Supplementary Figs. 5f and 5g, in which we noted a severe distortion. This distortion mainly originates from the thermal crosstalk (TC) between MRRs, which results in a severe degradation of the signal quality. Therefore, the TCC is essential to optimize the CDC.

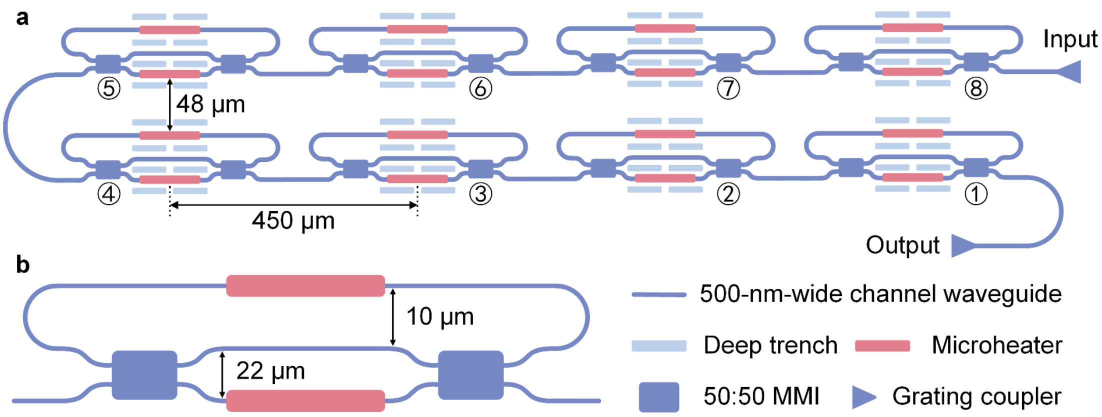

Supplementary Fig. 6. Schematic of the CDC. a Schematic layout of the 8 MRRs. The numbers label the sequence of MRRs. **b** Schematics of one MRR. Deep trenches are removed for a clear indication of the distance between waveguides. MMI: multimode interferometer.

Supplementary Fig. 6a shows the schematic layout of the CDC. The distances between the adjacent MRRs in the row and column directions are 450 μm and 48 μm, respectively. One MRR is shown in Supplementary Fig. 6b with the deep trenches removed for a better indication of the distances between the waveguides. The two arms of the MZI are separated by 22 μm, and the upper arm is 10-μm away from the straight waveguide in the MRR. There are mainly two types of TCs in the layout of the 8 MRRs. One is the intra-TC, which originates from the two phase shifters inside one MRR, and the other is the inter-TC, which comes from the TC between the MRRs.

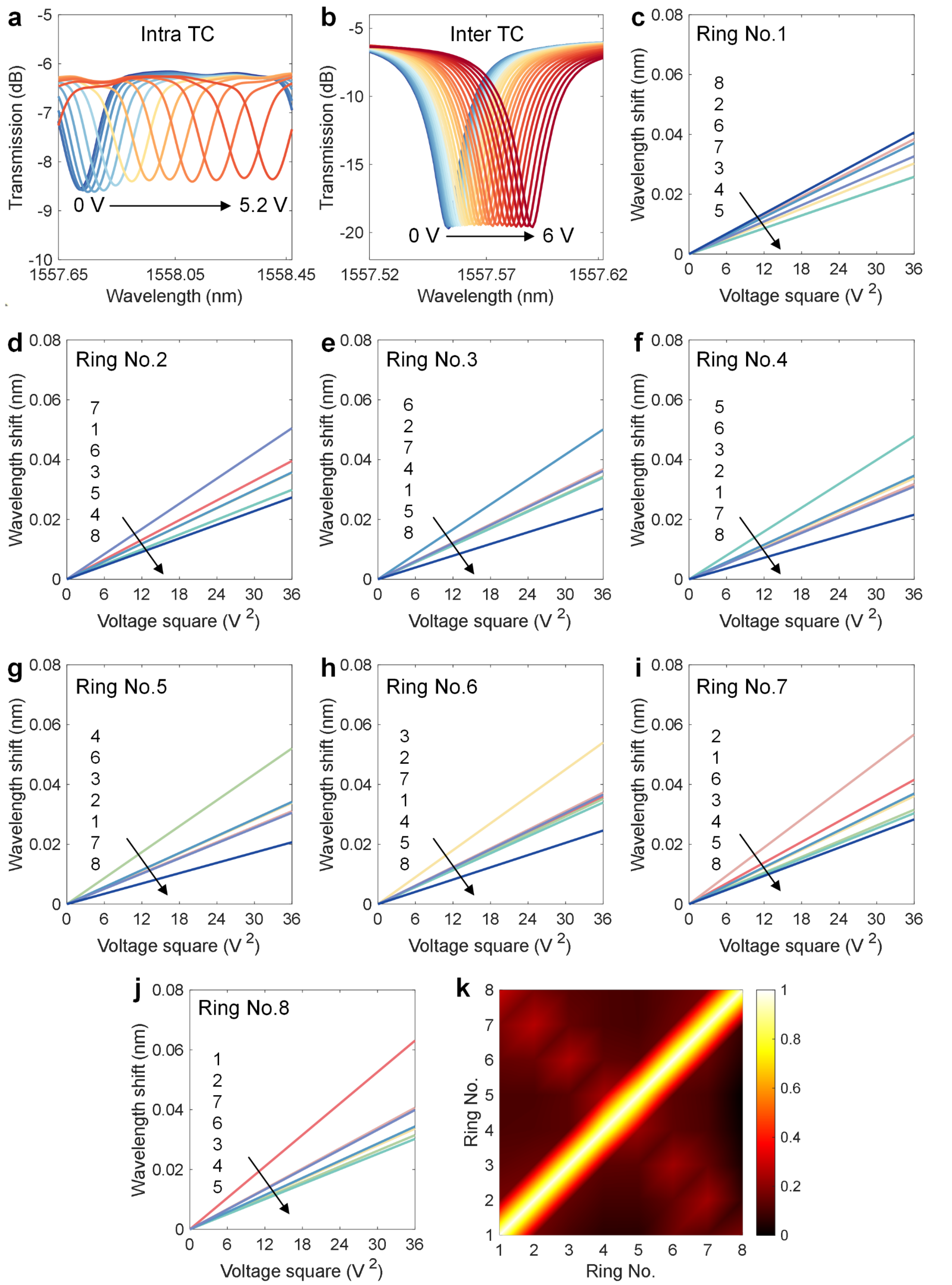

Supplementary Fig. 7. Characterizations of intra- and inter-TC of the CDC. a Measured transmission spectra of the first MRR when the wavelength-tuning phase shifter is applied with voltages. **b** Measured transmission spectra of the third MRR when the wavelength-tuning phase shifter of the second MRR is applied with voltages. **c-j** Measured resonant wavelength shift of the **(c)** first, **(d)** second, **(e)** third, **(f)** fourth, **(g)** fifth, **(h)** sixth, **(i)** seventh, and **(j)** eighth MRR when the resonant wavelength-tuning phase shifters of the other MRRs (indicated by labeled numbers) are applied with voltages. The MRR with the number on the top has the severest inter-TC to the MRR under test. **k** Normalized thermal crosstalk matrix of 8 MRRs.

For the intra-TC, as the MZI coupler is a part of the MRR, the coupling tuning in one arm of the MZI coupler intrinsically shifts the resonant wavelength. We calibrated this resonant wavelength shift during the over-coupling calibration process as the resonant wavelengths are fixed at the same wavelengths under the over-coupling regime. Therefore, the intra-TC mainly indicates whether the coupling is influenced by the tuning of the resonant wavelength of the same MRR. As all the MRRs share the same design, we chose one of them for the analysis. Supplementary Fig. 7a shows the measured transmission spectra of the first MRR with V_{1-res} swept from 0 V to 5.2 V with a step of 0.4 V. We tuned the MRR to the over-coupling regime and observed no evident coupling deviation, indicating that the intra-TC from the resonance tuning is negligible. As a result, we ignore the thermal influence of other MRRs on the coupling of the MRR under test as the phase shifters of the other MRRs are located even further away.

Therefore, we only need to consider the influence of inter-TC to the resonant wavelength shift of one MRR when tuning the resonant wavelengths and the coupling coefficients of all the other MRRs. Supplementary Fig. 7b shows the measured transmission spectra of the third MRR when V_{2-res} was swept from 0 V to 6 V with a step of 0.2 V. The resonant wavelength red-shifts with an increased V_{2-res}. Although a wavelength shift of only 0.04 nm was observed under a 6-V applied voltage, this inter-TC must be carefully considered because the overall performance of our CDC is sensitive to the resonant wavelength of each MRR. Therefore, we swept V_{i-res} $(i = 1, 2, \dots, 8)$ one by one, and obtained the corresponding transmission spectra. Supplementary Figs. 7c to 7j show the linearly fitted wavelength shift of the ith MRR when V_{j-res} $(j = 1, 2, \dots, 8,\ j \neq i)$ changes from 0 to 6 V. Here we take the first MRR for example. The impact of the inter-TC is sorted as 8>2>6>7>3>4>5, meaning that the 8th MRR has the most severe inter-TC to the 1st MRR and the 5th MRR has the mildest impact. To precisely characterize the influence of inter-TC, we extracted the slope of each line and obtained a thermal crosstalk matrix (TCM) as follows:

$$\mathrm{T} = 10^{-2} \cdot \begin{pmatrix} 2.5600 & 0.1070 & 0.0841 & 0.0718 & 0.0716 & 0.1030 & 0.0908 & 0.1130 \\ 0.1100 & 2.4200 & 0.0988 & 0.0830 & 0.0831 & 0.0993 & 0.1400 & 0.0760 \\ 0.0948 & 0.1020 & 2.4000 & 0.0954 & 0.0942 & 0.1390 & 0.1010 & 0.0655 \\ 0.0869 & 0.0885 & 0.0938 & 2.4200 & 0.1330 & 0.0962 & 0.0861 & 0.0599 \\ 0.0861 & 0.0862 & 0.0939 & 0.1450 & 2.3900 & 0.0949 & 0.0848 & 0.0574 \\ 0.0998 & 0.1030 & 0.1500 & 0.0977 & 0.0943 & 2.5500 & 0.1020 & 0.0683 \\ 0.1150 & 0.1580 & 0.1000 & 0.0876 & 0.0844 & 0.1030 & 2.3500 & 0.0786 \\ 0.1750 & 0.1130 & 0.0935 & 0.0874 & 0.0839 & 0.0955 & 0.1110 & 2.3900 \end{pmatrix} \tag{8}$$

All the elements of the TCM have a unit of nm/V^2, meaning that the resonant wavelength of the ith MRR red-shifts T_{ij}-nm when 1 V is applied on the jth MRR, where T_{ij} represents the matrix element on the ith row and the jth column. The diagonal matrix elements represent the resonant wavelength tuning efficiency of the ith MRR while the other elements are the inter-TC efficiencies. The normalized TCM is graphically shown in Supplementary Fig. 7k. The different levels of inter-TC demonstrate that the TC is the most severe between the MRRs located in the same column while it reduces when the MRRs are separated far away in the layout. We can readily understand this phenomenon as the micro-heater tends to heat up more along the longer side than along the shorter side, and the heat diffuses with length. We characterized the impact of the inter-TC when the couplings of other MRRs were tuned and found that it is almost identical to the one caused by the wavelength tuning, which we attribute to the close placement of the coupling-tuning and the wavelength-tuning phase shifters. Therefore, we can utilize the obtained TCM for the compensation of the inter-TC induced by the coupling tuning.

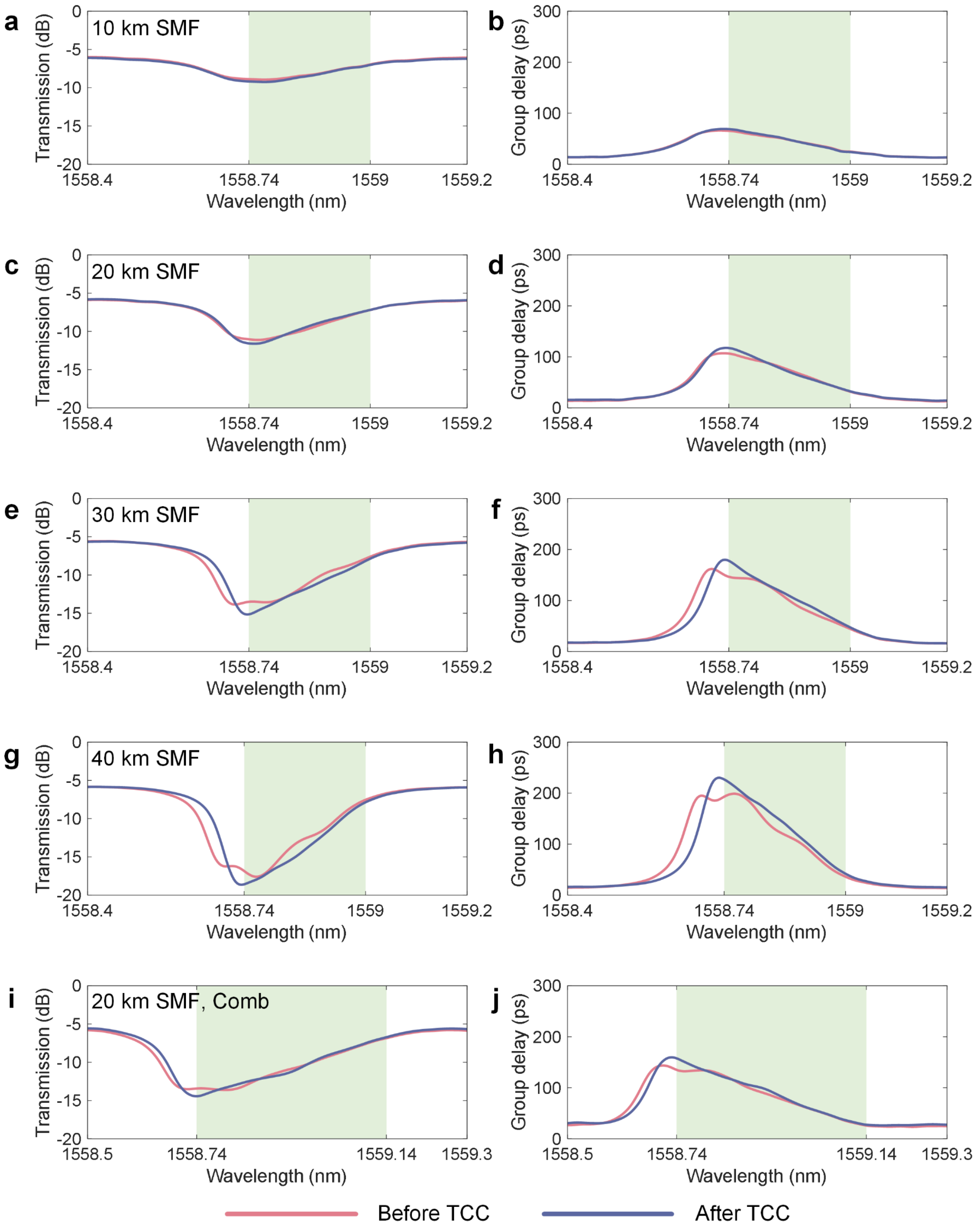

Supplementary Fig. 8. Measured transmission and group delay responses before and after the TCC. a-h Measured transmission spectra and group delay responses of CD compensation within 32-GHz bandwidth for **(a, b)** 10 km, **(c, d)** 20 km, **(e, f)** 30 km, and **(g, h)** 40 km SMFs transmission before and after the TCC. **i, j** Measured **(i)** transmission spectra and **(j)** group delay responses of CD compensation within 50-GHz bandwidth for 20-km SMF transmission before and after the TCC.

Finally, with the obtained TCM, we can compensate for the TC-induced wavelength shift and obtain the correct CD compensation state. As the TC has a negligible influence on the coupling coefficient of the MRRs, the applied voltages for the coupling tuning $V_{i-cpl}\ (i = 1, 2, \ldots, 8)$ are directly obtained according to the data shown in Supplementary Fig. 5e. To compensate for the influence of the inter-TC, we modify the applied voltage to the resonant wavelength-tuning phase shifter of the ith MRR as:

$$V_{i-final} = \sqrt{V_{i-res}^2 - \sum_{1\le j\le 8, i\ne j}\left(\frac{T_{ij}V_{j-res}^2}{FSR_i}\cdot V_{i_{2\pi}}^2\right) - \sum_{1\le j\le 8, i\ne j}\left(\frac{T_{ij}\left(V_{j_{DC}}^2 - V_{j_{ZC}}^2\right)}{FSR_i}\cdot V_{i_{2\pi}}^2\right)} \quad (9)$$

where $V_{i_{2\pi}}$ is the 2π-phase shift voltage of the ith MRR, and $V_{j_{ZC}}$ and $V_{j_{DC}}$ represent the applied voltages of the zero-coupling regime and of the desired-coupling regime of the jth MRR, respectively. The applied voltages before and after the TCC are demonstrated in Supplementary Table 3. The power consumption for reconfiguration of the CDC is less than 160 mW.

Supplementary Table 3. Applied voltages before and after the TCC

		Length of SMF (km)	MRR No. 1	2	3	4	5	6	7	8
**Coupling-tuning voltage (V)**		10	3.925	3.825	3.725	3.900	6.050	5.725	6.150	6.050
		20	4.200	3.525	3.375	3.575	3.700	5.725	6.150	6.050
		30	4.325	3.350	3.175	3.350	3.450	3.075	3.550	6.050
		40	4.400	3.200	2.950	3.125	3.200	2.800	3.350	3.450
		20 (Comb)	4.300	3.350	3.175	3.325	3.425	3.050	3.525	3.700
**Wavelength-tuning voltage (V)**	Before the TCC	10	4.823	5.360	3.716	3.803	0	0	0	0
		20	4.698	5.420	3.672	3.529	4.958	0	0	0
		30	4.636	5.443	3.574	3.363	4.699	4.841	2.807	0
		40	4.608	5.475	3.595	3.346	4.683	4.715	2.493	4.790
		20 (Comb)	4.666	5.435	3.653	3.452	4.834	4.800	3.039	5.953
	After the TCC	10	4.858	5.365	3.662	3.771	0	0	0	0
		20	4.760	5.441	3.635	3.501	5.015	0	0	0
		30	4.809	5.581	3.675	3.470	4.847	4.997	2.881	0
		40	4.834	5.659	3.764	3.516	4.875	4.915	2.654	4.920
		20 (Comb)	4.751	5.525	3.699	3.496	4.941	4.906	3.024	6.013

The transmission and the group delay responses before and after the TCC for the CD compensation for the 10-, 20-, 30-, and 40-km-long SMFs in a 32-GHz bandwidth and for the 20-km-long SMF in a 50-GHz bandwidth are illustrated in Supplementary Figs. 8a to 8j. Compared with the results without the TCC, the transmission and the group delay responses show much less distortion and exhibit a superior linearity within the operation bandwidth after the TCC. The impact of the TC is larger for the CD compensation with more utilized MRRs, which is because more TC is introduced with more utilized MRRs. We note that although some other TCC approaches based on thermal eigenmode decomposition have been proposed in the literature[3, 4], iterations are required to evaluate the exact elements of the TCC matrix. However, no iterations are required based on our TCC method, which simplifies calibration of the MRR-based CDCs. This method is applicable for other integrated photonic circuits based on thermal phase tuning. All the calibration tests were accomplished by software in our experiments.

Supplementary Note 4: 64-Gbit/s PAM4 data transmission using a CW-laser

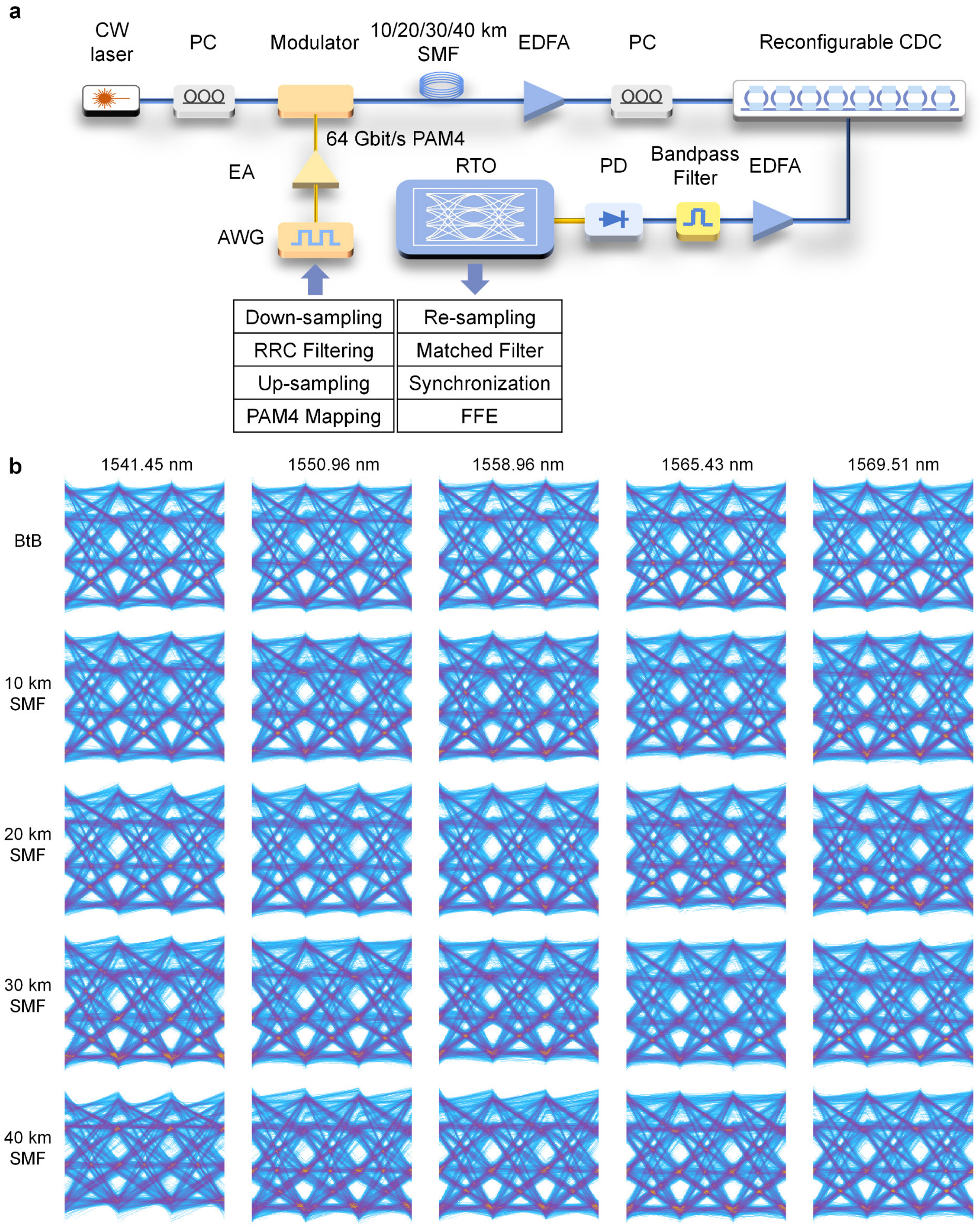

Supplementary Fig. 9. CW-laser-based data transmission. a Experimental setup schematics of signal transmission for CD compensation for 10-, 20-, 30-, and 40-km-long SMFs using a CW laser as the light source. CW: continuous-wave; PC: polarization controller; EDFA: erbium-doped fiber amplifier; EA: electrical amplifier; CDC: chromatic dispersion compensator; AWG: arbitrary waveform generator; PD: photodetector; RTO: real-time oscilloscope; RRC: root-raised cosine; FFE: feed-forward equalization. **b** Eye diagrams for BtB and transmissions with CD compensation under various wavelengths and lengths of SMFs.

The experimental setup schematics of the signal transmissions with CD compensation for 10-, 20-, 30-, and 40-km-long SMFs in a 32-GHz bandwidth under 5 different wavelengths in the C-band is shown in Supplementary Fig. 9a. The wavelengths of 1541.45 nm, 1550.96 nm, 1558.96 nm,

1565.43 nm, and of 1569.51 nm were chosen in the experiment. All the wavelengths were arbitrarily chosen except for 1558.96 nm, at which the CDC was calibrated. The output power from the continuous-wave (CW) laser was maintained at 12.5 dBm for all the measurements. A pulse-amplitude four-level modulation (PAM4) signal generated by an arbitrary waveform generator (AWG, Keysight 8199A) with a maximum sampling rate of 256 GSa/s was amplified by an electrical amplifier and then applied to drive a 40-GHz-bandwidth commercial intensity modulator (iXblue MXAN-LN-40). After the transmission over the lengths of the SMFs, we amplified the modulated signal by an erbium-doped fiber amplifier (EDFA) to ensure that the power of the optical signal after the CDC was kept at about -5 dBm, which is intended to compensate for the non-uniform loss across a broad wavelength span introduced by the grating coupler. We utilized another EDFA to ensure the power of the received optical signal at the 50-GHz photodetector (PD, Finisar XPDV2320R) was maintained at around 3 dBm. We obtained the electrical waveforms using a real-time oscilloscope (RTO, Keysight Z592A) with a sampling rate of 80 GSa/s for offline digital signal processing (DSP).

The measured eye diagrams of the data transmission under back-to-back (BtB) conditions, and the transmissions with CD compensation for the 4 different lengths of the SMFs in a 32-GHz bandwidth under the 5 wavelengths are shown in Supplementary Fig. 9b. The eye diagrams are clear with open eyes in the BtB transmissions, and we obtained comparable ones after the CD compensation. The eye diagrams without CD compensation are shown in Fig. 2e in the manuscript. They are blurred with closed eyes without CD compensation.

Supplementary Note 5: 80-Gbit/s PAM4-based parallel data transmission

The experimental setup schematics of the microcomb-driven parallel PAM4 data transmission is shown in Fig. 3a of the manuscript. In the experiment, the 7th channel of the comb lines (1558.96 nm) was aligned with the center of the operation bandwidth of the CDC. We show the measured eye diagrams of all the 15 comb lines in the BtB and the CD-compensated transmissions in Supplementary Figs. 10a and 10b. Compared with the other eye diagrams, those of the 1st, 13th,14th, and 15th channels were slightly distorted owing to the FSR mismatch between the comb lines and the CDC.

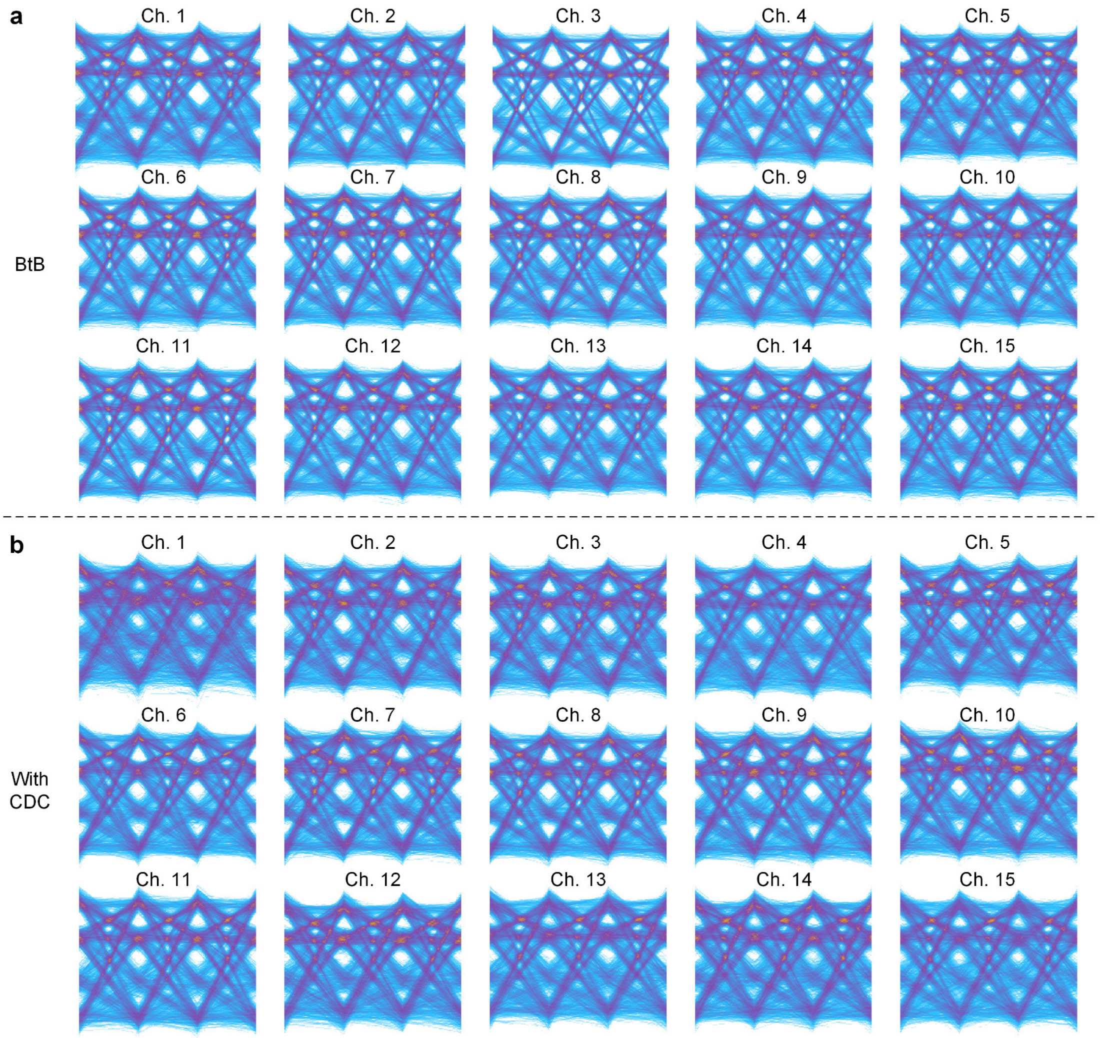

Supplementary Fig. 10. Microcomb-based 80-Gbit/s PAM4 parallel data transmission. a, b Eye diagrams of 15 comb lines using 80-Gbit/s PAM4 modulation signal under **(a)** BtB transmission and **(b)** transmissions with CD compensation. CDC: chromatic dispersion compensator.

Supplementary Note 6: 112-Gbit/s DMT-based parallel data transmission

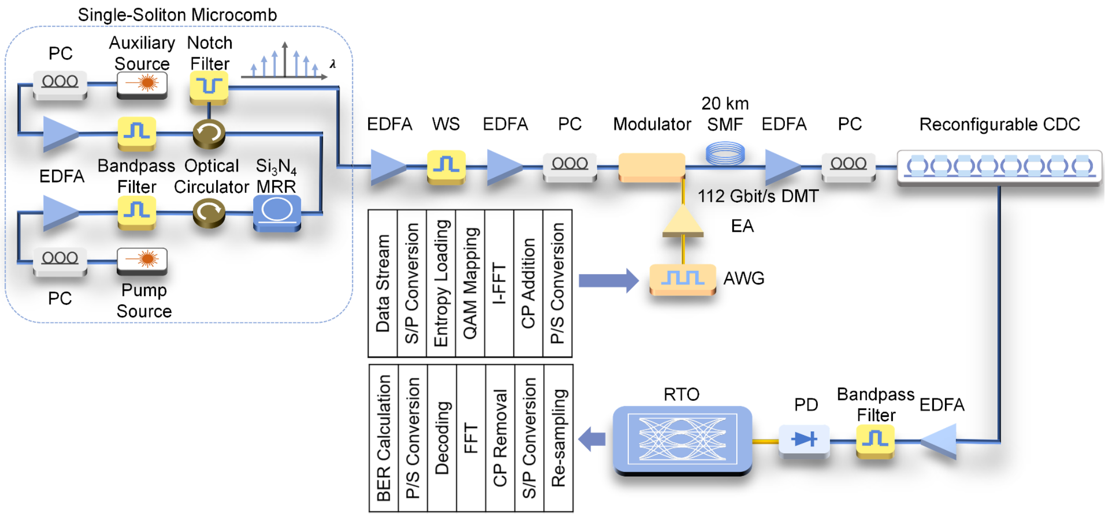

Supplementary Fig. 11. Experimental setup of the DMT parallel data transmission. P/S Conversion: parallel to serial conversion; S/P Conversion: serial to parallel conversion; FFT: fast Fourier transform; I-FFT: inverse fast Fourier transform; QAM: quadratic amplitude modulation; CP: cyclic prefix.

The experimental setup schematics of the 112-Gbit/s DMT-based parallel data transmission is shown in Supplementary Fig. 11, which is almost identical to that of the PAM4-based parallel data transmission except for the DSP procedures and for the modulation format. During the measurement, we obtained the constellations of the total 160 sub-carriers of each comb line, and those of the 7th sub-carrier, with a 16-QAM modulation format, of all the 15 comb lines after CD compensation are shown in Supplementary Fig. 12. The constellations are only slightly deteriorated after the fiber transmission, indicating an excellent CD compensation performance of our device.

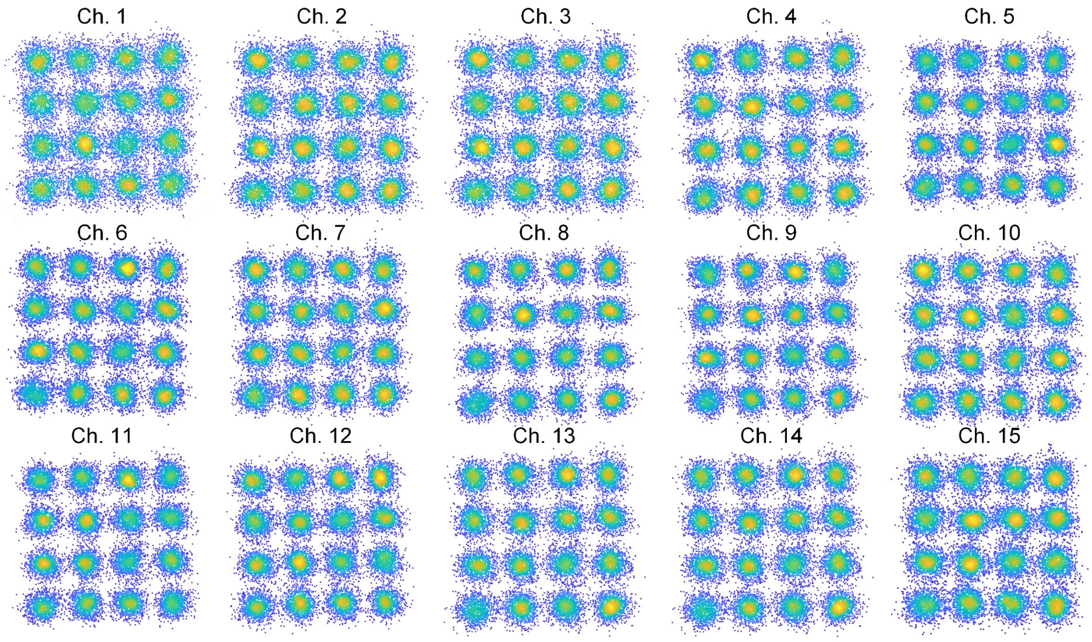

Supplementary Fig. 12. Microcomb-based 112-Gbit/s DMT parallel data transmission. Constellations for 16-QAM modulation of the 15 comb lines using a 112-Gbit/s DMT signal after the CD compensation for the 7th sub-carrier.

As for the bit rate of the DMT transmission, we calculate the raw bit rate by 64 GSa/s×297bits/(2×80GSa/s+10) = 112 Gbit/s, where 64 GSa/s and 80 GSa/s are the sampling rates of the AWG and of the RTO, 297 is the total number of bits of one DMT symbol, and 10 is the length of the cyclic prefix sequence. We transmitted a 112 Gbit/s DMT signal in each channel of the 15 comb lines, which leads to an aggregate total bit rate of 15*112 Gbit/s = 1.68 Tbit/s.

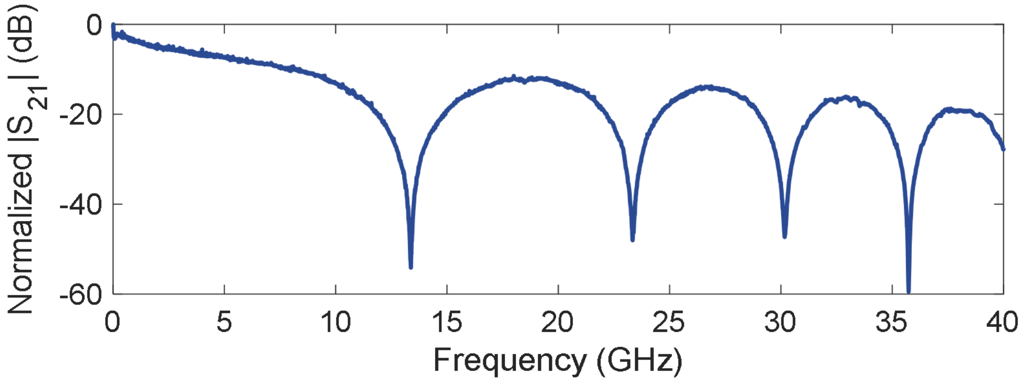

Supplementary Fig. 13. Measured normalized S_{21} magnitude response of the 20-km SMF. The FSF-induced response null points appear at the frequencies of around 13 GHz, 23 GHz, 30 GHz, and 36 GHz.

Supplementary Fig. 13 shows the measured S_{21} magnitude response of the 20-km SMF in a frequency span of less than 40 GHz. The CD-induced frequency-selective fading (FSF) leads to periodic null points at the frequencies of around 13 GHz, 23 GHz, 30 GHz, and 36 GHz, which accounts for the measured high bit-error rates (BERs) at the frequencies of 13 GHz and of 23 GHz without CD compensation in the experiments shown in Fig. 4d of the manuscript.

Supplementary Note 7: Power consumption calculation of CD compensation

In the following parts, we talk about the power consumptions of different CD compensation methods first, and then we make a wholistic comparison and discussion about the normalized power consumptions with the unit of pJ/bit for CDC of 40-km SMFs.

(i) The power consumption for DCFs. As DCFs are passive components and do not require any power for CD tuning, the power consumption of using DCFs for CD compensation mainly comes from the power amplifiers (typically EDFAs) that are used to compensate for the loss induced by the DCFs. The attenuation of the DCF by Thorlabs[5] is less than 0.265 dB/km, and the dispersion ranges from -49 ps/(nm·km) to -30 ps/(nm·km). We assume that the dispersion of SMF and DCF is 18 ps/(nm·km) and -36 ps/(nm·km), respectively, which means that a maximum of 60-km-long DCF is required for the compensation of CD of up to 120 km SMF. In this case, the DCF introduces a maximum insertion loss of 15.9 dB. We assume the 60 km DCF is followed by a 120 km SMF. We consider the output of the SMF to be connected with a receiver, and we assume a -10 dBm received optical power at the receiver end (In Ref 6, the author mentioned an around -10 dBm received optical power can achieve a BER below 1e-3). If we consider a 0.2 dB/km attenuation of the SMF, we can obtain the optical power at the input of the SMF to be $-10 + 0.2 \times 120 = 14\ dBm\ (\sim 25\ mW)$. After the introduction of DCF, the optical power reduces to $P_{DCF} = 14 - 15.9 = -1.9\ dBm\ (\sim 0.65\ mW)$. We use the EDFA to compensate the optical power from $P_{DCF} = -1.9\ dBm$ back to $P_{out} = 14\ dBm$. Then the power consumption induced by the EDFA can be calculated by:

$$PC_{EDFA} = (P_{out} - P_{DCF})/\eta_{wp} \tag{10}$$

where η_{wp} is the wall-plug efficiency of the EDFA. A 16% wall-plug efficiency is assumed here (We will discuss this efficiency in part (vi)). In this case, the estimated power consumption of the EDFA is ~152 mW. The power consumption of using DCF increases exponentially with the length of SMF. To be mentioned, the DCF can also be used for parallel CD compensation due to their broad optical bandwidth. And its colorless operation requires no temperature control. However, the DCF is bulky and can only compensate for the CD of a fixed length of the SMF. Therefore, we will not include the DCF in the power consumption comparison.

(ii) The power consumption of our CDC. The power consumption for controlling the states of CDC is below 160 mW, which can be calculated by referring to the applied voltages shown in Supplementary Table 3. As our CDC brings in an extra loss that should be compensated by an EDFA, we also consider the power consumption of the EDFA. For the parallel data transmission, we assume a uniform transmission loss of 18 dB of different channels for 20 km SMF transmission, including ~10 dB on-chip insertion loss at the center of the operation bandwidth (see Supplementary Fig. 8i) and ~8-dB fiber coupling loss from the grating couplers. In the experiment, the total optical power of the 15 comb lines before our CDC was maintained at $17.5\ dBm\ (\sim 56.23\ mW)$. After the introduction of our CDC, the total optical power decreased to $17.5 - 18 = -0.5\ dBm\ (\sim 0.89\ mW)$. We used an EDFA to compensate the total optical power back to 17.5 dBm after the CDC. The input optical power P_{in} and output optical power P_{out} of the EDFA is $-0.5\ dBm$ and $17.5\ dBm$, respectively. The power consumption of the EDFA PC_{EDFA} can be then calculated by

$$PC_{EDFA} = (P_{out} - P_{in})/\eta_{wp} \tag{11}$$

The PC_{EDFA} can then be estimated to be ~346 mW, leading to a total power consumption of less than 506 mW for our CDC. Thus, the power consumption of our CDC is ~0.3 pJ/bit (1.68 Tbit/s data

transmitted).

(iii) The power consumption of CD compensation with existing coherent optical transceiver modules. Inphi[7] (now Marvell) demonstrated a 400G ZR transceiver module with a power consumption of 4 W/100G. NeoPhotonics[8] (now Lumentum) and InnoLight[9] demonstrated 400G ZR modules that consume 18 W power for 400G transmission, respectively. The transmission length is less than 120 km and these power consumptions are generally for the entire module. To get the power consumption of the CD compensation, we assume that the DSP consumes about 50% of the module[7] and the CD compensation accounts for ~25% of the DSP power[10]. In this case, the power consumption of the DSP-based CD compensation of Inphi's 400 ZR modules is calculated as 4×50%×25%=0.5 W/100G. Thus, the estimated power consumption in units of pJ/bit of these three modules are 5 pJ/bit, ~5.6 pJ/bit, and ~5.6 pJ/bit for 120-km-SMFs CDC, respectively.

(iv) The power consumption of CD compensation with next-generation coherent transceiver modules. The maximum total power consumptions allowed for 800G ZR using Quad Small Form Factor Pluggable-Double Density (QSFP-DD)[11, 12] and Octal Small Form Factor Pluggable (OSFP)[12] form factors are 25 W and 30 W, respectively. Maximum total power consumption of 33 W is allowed for 1.6T ZR using OSFP-Extra Dense (OSFP-XD) form factor[12]. These modules are designed for the SMF transmission of less than 120 km. Using a similar calculation method, we can obtain that the estimated power consumption of CD compensation for 800G ZR coherent using QSDP-DD and OSFP is ~3.9 pJ/bit and ~4.7 pJ/bit, respectively. The power consumption of CD compensation for 1.6T ZR coherent is estimated to be ~2.6 pJ/bit.

(v) The normalized power consumption of CD compensation of various methods. As the mentioned CD compensation methods have different transmission lengths and the power consumption is larger for longer SMF transmission, we thus normalize the length of SMF to 40 km for a fair comparison. **(I)** For our own CDC, the increase in power consumption mainly originates from the power consumption of the EDFA, as the power consumption of our chip remains nearly unchanged for compensation of different SMF lengths. There is an extra loss of ~5 dB between the CD compensation of 40 km and 20 km SMF (see Fig. 2d in the manuscript, the loss is obtained at the center of the operation bandwidth), and the total power consumption of EDFA is calculated by $\frac{10^{\frac{17.5}{10}}-10^{\frac{17.5-18-5}{10}}}{0.16} = \sim 350$ mW. The total power consumption is then ~510 mW, leading to a normalized one of ~0.3 pJ/bit. **(II)** For the coherent transceiver modules, we assume that the power consumption of CD compensation varies almost linearly with the SMF length of less than 120 km according to the data in Fig. 12 of Ref 7. Therefore, the power consumption for CD compensation of 40 km SMF is one-third of that for 120 km transmission, resulting in a normalized power consumption of ~1.7 pJ/bit, ~1.9 pJ/bit, and ~1.9 pJ/bit for the Inphi, NeoPhotonics, and InnoLight transceiver modules, respectively. The normalized power consumptions are estimated to be ~1.3 pJ/bit (800G ZR QSFP-DD), ~1.6 pJ/bit (800G ZR OSFP), and ~0.9 pJ/bit (1.6T ZR OSFP-XD) for the next generation DCI applications.

(vi) Wall-plug efficiency. A power efficiency of more than 80% from the pump laser diodes to the EDFA output has been demonstrated decades ago[13]. In addition, many laser diodes pumped at 1480 nm can achieve a power conversion efficiency of more than 20%, like the one from SemiNex[14]. Therefore, we conservatively estimate that the wall-plug efficiency of EDFA is 16% (80%×20%), and the power consumption of EDFA only includes the one of the pump laser diodes.

We list the performances and the normalized power consumptions of different CD compensation

methods in Supplementary Table 4. We can see that the estimated power consumption of our CDC is about 6 times less than that of the existing 400G ZR coherent transceiver modules, and is even about 3 times less than that of the next-generation 1.6T ZR coherent modules. Based on the analysis, we can see that our work is remarkably competitive with the existing or even next generation commercial modules in terms of power consumption, and is suitable for the DCI applications where the power consumption is of vital importance.

Supplementary Table 4. Comparison of various CD compensation methods

Methods	This work	Coherent (400G ZR)			Coherent (800G/1.6T ZR, Next Generation)		
Company/ Form factor	/	Marvell[7]	Lumentum[8]	InnoLight[9]	QSFP-DD[11, 12]	OSFP[12]	OSFP-XD[12]
DSP node	/	7 nm	7 nm	7 nm	5 nm	5 nm	3 or 2 nm
Transmission range (km)	0~40	<120					
Total data rate (Gbit/s)	1680	400	400	400	800	800	1600
Power consumption (pJ/bit)	~0.3	~1.7	~1.9	~1.9	~1.3	~1.6	~0.9

We further show how the power consumption increases with the length of SMF in Fig. 5a in the manuscript. For the CD compensation of 40 km SMF, we consider the power consumption to be consistent with the previous analysis, in which 160 mW is from our CDC, and 350 mW is from EDFA. An extra 7 MRRs should be introduced for the CD compensation of an additional 40 km SMF, leading to a 140 mW power consumption increment from the CDC, assuming that each MRR consumes an average power consumption of 20 mW. Thus, the power consumption of CDC for the CD compensation of 40 km, 80 km, and 120 km SMF is 160 mW, 300 mW, and 440 mW, respectively. In addition, we assume a 17.5 dBm total optical input power of our CDC, and a 10-dB extra loss (due to the additional MRRs, obtained from measurement results in Fig. 2d in the manuscript) is introduced for every extra 40 km SMF. We consider using an additional EDFA to compensate for the loss for every extra 40 km SMF transmission, similar to the schematic shown in Fig. 1 in Ref 15. Then the power consumption of EDFA for CD compensation of 40 km, 80 km, and 120 km SMF is 350 mW, 666 mW, and 982 mW, respectively. In this case, the power consumption of our CDC is estimated to be ~0.3 pJ/bit, ~0.6 pJ/bit, and ~0.8 pJ/bit. For the existing 400G ZR coherent, we also assume the power consumption of CD compensation varies almost linearly with the length of SMF, and the corresponding power consumption of 40 km, 80 km, and 120 km CD compensation is ~1.7 pJ/bit, ~3.3 pJ/bit, and 5 pJ/bit, respectively. We can see that the estimated power consumption of our CDC is around 6 times less and increases much slower than the coherent DSP with an increased SMF length.

The relationship between the power consumption and the total data rate is shown in Fig. 5b in the manuscript. We consider a total data rate of 400 Gbit/s, 800 Gbit/s, 1.6 Tbit/s, 3.2 Tbit/s, 6.4 Tbit/s, 11.424 Tbit/s, and 12.8 Tbit/s. The total input optical power in our work is 17.5 dBm, and we used 15 comb lines, resulting in an average optical power of ~5.74 dBm (~3.75 mW). The number

of the used comb lines is 4, 8, 15, 29, 58, 102, and 115 for 400 Gbit/s, 800 Gbit/s, 1.6 Tbit/s, 3.2 Tbit/s, 6.4 Tbit/s, 11.424 Tbit/s, and 12.8 Tbit/s data rate, leading to a total optical input power of ~11.76 dBm, ~14.77 dBm, 17.50 dBm, ~20.36 dBm, ~23.37 dBm, ~25.83 dBm, and ~26.35 dBm, respectively. We also consider an 18 dB loss of our CDC (20 km) and consider an extra 5 dB loss of CD compensation of 40 km SMF compared to the one of 20 km. The power consumption of our chip is fixed at 160 mW. We take the calculation of the power consumption for 400 Gbit/s data rate as an example. The power consumption can be calculated by $\frac{10^{\frac{11.76}{10}}-10^{\frac{11.76-18-5}{10}}}{0.16}+160=\sim 253$ mW. Using a similar method, the total power consumption of our CDC under the seven scenarios is estimated to be 0.253 W, 0.347 W, 0.510 W, 0.836 W, 1.512 W, 2.538 W, and 2.841 W, respectively, while the one of coherent DSP is 0.68 W, 1.36 W, 2.72 W, 5.44 W, 10.88 W, ~19.42 W, and 21.76 W, respectively. The estimated power consumption of the coherent modules maintains at 1.7 pJ/bit, while it is ~0.6 pJ/bit, ~0.4 pJ/bit, ~0.3 pJ/bit, ~0.3 pJ/bit, ~0.2 pJ/bit, ~0.2 pJ/bit, and ~0.2 pJ/bit for our CDC. Our CDC exhibits a much smaller total power consumption when the total transmitted data rate is getting larger, and the normalized power consumption is even lower for higher data rates.

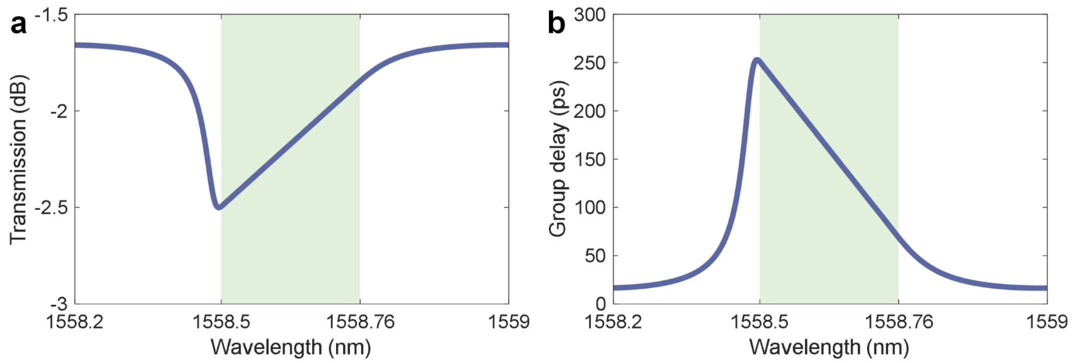

Supplementary Fig. 14. Transmission and group delay characteristics of the CD compensation with improved propagation loss. a, b Simulated **(a)** transmission and **(b)** group delay response of the CD compensation of 40 km SMF, assuming a 0.5 dB/cm propagation loss of the MRR.

Furthermore, we consider an optimized coupling loss of our CDC and a low insertion loss of our chip (~0.5 dB/cm), and then further analyze the power consumption. The authors in Ref 16 experimentally demonstrated a coupling loss of ~0.32 dB/facet in 2015, which is relatively the smallest one for coupling. The total loss of our CDC for the CD compensation of 20 km SMF is then 10.64 dB (10 dB insertion loss and 0.64 dB coupling loss). We calculate the power consumption using a similar method. The power consumptions of CD compensation for various SMF lengths and various data rates are listed in Supplementary Table 5 and shown in Fig. 5 in the manuscript. In addition, we assume a low waveguide loss of 0.5 dB/cm and a ~0.1 dB insertion loss for each MMI. Supplementary Figs. 14a and 14b show the simulated transmission and group delay response of the CD compensation for 40 km SMF. We can obtain an insertion loss of ~2.2 dB from the center of the operation bandwidth. We then consider an insertion loss of ~2.2 dB and a coupling loss of ~0.64 dB. An extra insertion loss of 2.2 dB is considered for an additional 40 km SMF. Extra 7 MRRs are also considered for the CD compensation of an additional 40 km SMF.

We obtain the normalized power consumption for the CD compensation of various SMF lengths and various data rates, as shown in Supplementary Table 5 and Fig. 5 in the manuscript. We can see that the normalized power consumptions of low coupling loss (CL) are almost identical to the ones in our experiment. There is a power consumption reduction of ~0.1 pJ/bit, ~0.2 pJ/bit, and ~0.3

pJ/bit for 40 km, 80 km, and 120 km SMF transmissions if low CL and low insertion loss (IL) are assumed, respectively, corresponding to a reduction of ~35.5%, ~37.0%, and ~37.5%. The estimated power consumption of our CDC for higher data rate is even lower, and under the 11.424 Tbit/s data transmission rate, it is around 15 times less than that of the coherent DSP if low CL and low IL are assumed.

Supplementary Table 5. Calculated power consumption of various scenarios

Scenarios		Coherent DSP (pJ/bit)	MRR-based CDC (pJ/bit)	MRR-based CDC with low CL (pJ/bit)	MRR-based CDC with low CL and IL (pJ/bit)
Various SMF lengths (km)	40	1.7	~0.3	~0.3	~0.2
	80	3.3	~0.6	~0.6	~0.4
	120	5	~0.8	~0.8	~0.5
Various data rate (Gbit/s)	400	1.7	~0.6	~0.6	~0.5
	800	1.7	~0.4	~0.4	~0.3
	1600	1.7	~0.3	~0.3	~0.2
	3200	1.7	~0.3	~0.3	~0.2
	6400	1.7	~0.2	~0.2	~0.1
	11424	1.7	~0.2	~0.2	~0.1
	12800	1.7	~0.2	~0.2	~0.1

Supplementary Note 8: Scalability of the transmission system

In the parallel data transmission experiment, the operation bandwidth of our CDC is tuned to 50 GHz, while the transmitted data rate per channel is limited to 80 Gbps for the PAM4 modulation format, which is due to the limited bandwidth of the intensity modulator (~32-GHz 3-dB bandwidth). We measured eye diagrams of a 45 Gbaud PAM4 signal, and they were blurred even under the BtB condition using a CW laser as the light source. Thus, to scale the transmission system to achieve a total bit rate exceeding 10 Tbit/s, we prefer to design the microcomb and the CDC with a narrower spacing of 50 GHz.

As illustrated in the discussion in the manuscript, the number of the available comb lines is 51 for our existing microcomb, and it can be doubled if the microcomb has an FSR of 50 GHz. To achieve a total bit rate of more than ten terabits, each channel should at least transmit a data rate of 100 Gbit/s. Compared with the PAM4, the modulation format of DMT is preferred as it has a higher spectral efficiency. In our experiment, the utilized bandwidth of the 112 Gbit/s DMT signal is around 40 GHz, indicating that the operation bandwidth of the CDC should be at least 40 GHz. We note that this operation bandwidth can be achieved with 8 cascaded MRRs. Supplementary Fig. 15 shows the simulated transmission and group delay responses of the 8 MRRs for the CD compensation of 20 km SMF. A waveguide loss of 4 dB/cm is assumed. Therefore, we can achieve a total data rate of 11.424 Tbit/s by employing 102 comb lines.

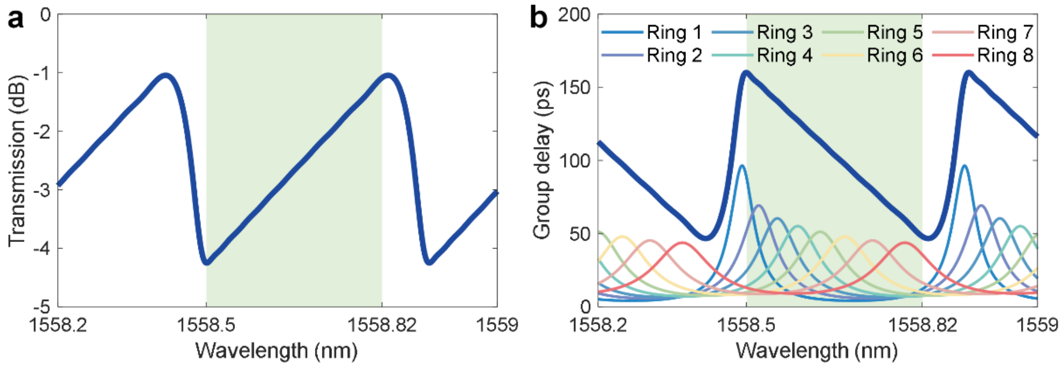

Supplementary Fig. 15. Transmission and group delay characteristics of the MRRs with a 50-GHz FSR. **a, b** Simulated **(a)** transmission and **(b)** group delay responses for the CD compensation of 20 km SMF for MRRs with a 50-GHz FSR.

Supplementary Note 9: The required number of MRRs and the corresponding power consumption for CD compensation of various fiber lengths and operation bandwidth

Supplementary Table 6 summarizes the required number of MRRs for various operational bandwidth and fiber length. We consider a fiber length of 40 km, 60 km, and 80 km, and an operation bandwidth of 40 GHz, 50 GHz, and 60 GHz. We assume an 18 ps/(nm·km) dispersion of the SMF. Supplementary Fig. 16 shows the simulated transmission and group delay responses for the listed conditions of 40 km, 60 km, and 80 km SMF transmission. We can see that 14 MRRs are required for the CD compensation of 40 km SMF within a bandwidth of 50 GHz. In addition, we also simulate the CD compensation of 100 km and 120 km SMF but with a smaller operation bandwidth. We need 15 and 17 MRRs to achieve the CD compensation of 100 km and 120 km SMF within a bandwidth of 32 GHz, respectively. We can infer from the simulations that extra 4 MRRs, 6 MRRs, and 8 MRRs are required for an extra 10 GHz operational bandwidth for CD compensation of 40 km, 60 km, and 80 km SMF, respectively. And we can also deduce the number of MRRs for CD compensation of 100 km SMF under various bandwidth. In this case, we need 26 MRRs to realize the CD compensation for 40 km with a bandwidth of 80 GHz. We should note that we can halve the FSR of the microcomb and the CDC to achieve a total data rate of larger than 10 Tbit/s while maintaining the number of MRRs at 8, as discussed in the Supplementary Note 8. In this case, we do not need to use too many MRRs as shown in the Supplementary Table 6.

Supplementary Table 6. Number of MRRs required for different bandwidth and fiber lengths

**Number of MRRs**		**Length of SMF (km)**				
		**40**	**60**	**80**	**100**	**120**
**Operation bandwidth (GHz)**	32	/	/	/	15	17
	40	10	13	17		
	50	14	19	25		
	60	18	25	33		
	70 (Deduced)	22	31	41	/	
	80 (Deduced)	26	37	49		

In our work, the measured power consumption is 160 mW for 8 MRRs, corresponding to an average power consumption of 20 mW for each MRR. If we only consider the power consumption of the MRRs, Supplementary Fig. 17 shows how the calculated power consumption increases with the bandwidth and fiber length. The power consumption increases linearly with the operational bandwidth under various lengths of SMF. And the increment of power consumption increases linearly with lengths of SMF.

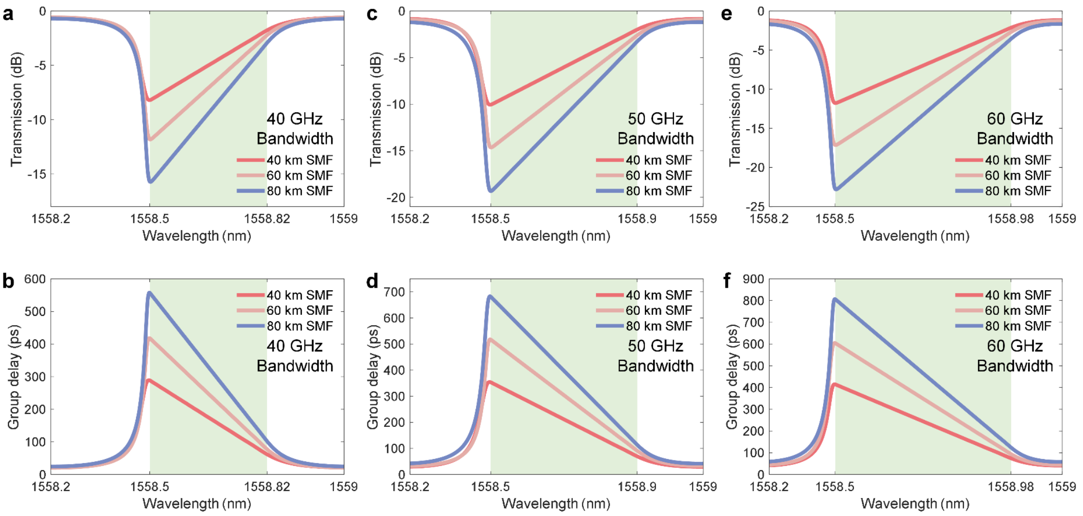

Supplementary Fig. 16. Simulated transmission and group delay responses for various operation bandwidth and fiber length. a, c, e Simulated transmission responses for an operational bandwidth of **(a)** 40 GHz, **(c)** 50 GHz, and **(e)** 60 GHz for CD compensation of 40 km, 60 km, and 80 km SMF. **b, d, f** Simulated group delay responses for an operational bandwidth of **(b)** 40 GHz, **(d)** 50 GHz, and **(f)** 60 GHz for CD compensation of 40 km, 60 km, and 80 km SMF.

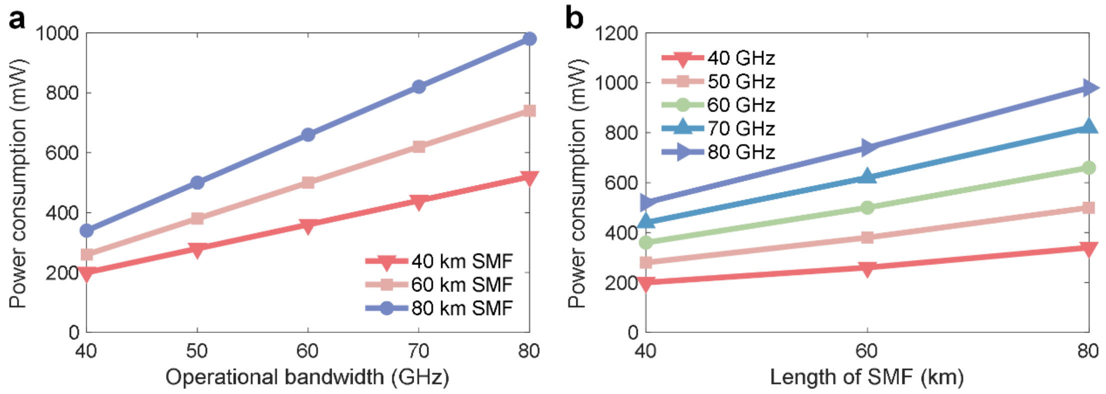

Supplementary Fig. 17. Power consumption of the CDC for different operational bandwidth and lengths of SMF. a Power consumption of the CDC for different operational bandwidth under various lengths of SMF. **b** Power consumption for different lengths of SMF under different operational bandwidth.

References

1. Zhang HY, Lu LJ, Chen JP, Zhou LJ. Program-Controlled Single Soliton Generation Driven by the Thermal-Compensated Avoided Mode Crossing. *Journal of Lightwave Technology* **41**, 1801-1810 (2023).
2. Fujii S, Tanabe T. Dispersion engineering and measurement of whispering gallery mode microresonator for Kerr frequency comb generation. *Nanophotonics* **9**, 1087-1104 (2020).
3. Milanizadeh M, Aguiar D, Melloni A, Morichetti F. Canceling Thermal Cross-Talk Effects in Photonic Integrated Circuits. *Journal of Lightwave Technology* **37**, 1325-1332 (2019).
4. Milanizadeh M*, et al.* Control and Calibration Recipes for Photonic Integrated Circuits. *IEEE Journal of Selected Topics in Quantum Electronics* **26**, 1-10 (2020).
5. Thorlabs. Dispersion compensating fibers, https://www.thorlabs.com/catalogpages/obsolete/2017/DCF38.pdf.
6. Zhou X, Urata RH, Liu H. Beyond 1 Tb/s Intra-Data Center Interconnect Technology: IM-DD OR Coherent? *Journal of Lightwave Technology* **38**, 475-484 (2020).
7. Nagarajan R, Lyubomirsky I, Agazzi O. Low Power DSP-Based Transceivers for Data Center Optical Fiber Communications (Invited Tutorial). *Journal of Lightwave Technology* **39**, 5221-5231 (2021).
8. Lumentum. 400G ZR Coherent transceiver, https://www.lumentum.com/en/products/400g-zrzr-qsfp-dd-dco.
9. InnoLight. 400G ZR Coherent transceiver, https://www.innolight.com/en/goods/solution/cid/16.html.
10. Seiler PM*, et al.* Toward coherent O-band data center interconnects. *Frontiers of Optoelectronics* **14**, 414-425 (2021).
11. Dogruoz B*, et al.* Optimizing QSFP-DD Systems to Achieve at Least 25 Watt Thermal Port Performance, http://www.qsfp-dd.com/wp-content/uploads/2021/01/2021-QSFP-DD-MSA-Thermal-Whitepaper-Final.pdf (2021).
12. Tauber D*, et al.* Role of Coherent Systems in the Next DCI Generation. *Journal of Lightwave Technology* **41**, 1139-1151 (2023).
13. Massicott JF, Wyatt R, Ainsile BJ, Crag-Ryan SP. Efficient, high power, high gain, Er3+ doped silica fibre amplifier. *Electronics letters* **26**, 1038-1039 (1990).
14. https://seminex.com/product/chip-laser-diode-8/.
15. Pillai BSG, Sedighi B, Shieh W, Tucker RS. Chromatic dispersion compensation — An energy consumption perspective. In: *Optical Fiber Communication Conference and Exhibition/National Fiber Optic Engineers Conference (OFC/NFOEC)* (2012).
16. Cheben P*, et al.* Broadband polarization independent nanophotonic coupler for silicon waveguides with ultra-high efficiency. *Optics Express* **23**, 22553-22563 (2015).
